# Association between alcohol consumption pattern and the incidence risk of type 2 diabetes in Korean men: A 12-years follow-up study

**DOI:** 10.1038/s41598-017-07549-2

**Published:** 2017-08-04

**Authors:** Dae-Yeon Lee, Min-Gyu Yoo, Hyo-Jin Kim, Han Byul Jang, Jae-Hong Kim, Hye-Ja Lee, Sang Ick Park

**Affiliations:** 1 Center for Biomedical Sciences, Korea National Institute of Health, Cheongju, 28159 Korea; 2 School of life science and biotechnology, Korea University, Seoul, 02841 Korea

## Abstract

Moderate alcohol consumption is generally associated with reduced risk of type 2 diabetes. However, this beneficial effects of alcohol intake remains controversial due to inconsistent results across studies. The analysis was performed using data from the Ansung-Ansan cohort study. We categorized the participants into four groups—based on the baseline (one-point measure; non-drinking, <5 g/day, ≥5, <30 g/day, and ≥30 g/day) and follow-up (consumption pattern; never-drinking, light, moderate, and heavy drinking) measurement. At baseline, ≥30 g/day alcohol consumption increased the risk of incident diabetes (HR: 1.42; 95% CI, 1.10–1.85), but ≥5, <30 g/day alcohol consumption had no effects on the incident diabetes. Meanwhile, when using the alcohol consumption pattern, a heavy-drinking pattern increased the risk of incident diabetes (HR = 1.32, 1.01–1.73), but the light and moderate consumption pattern was associated with a reduced risk of type 2 diabetes (HR: 0.66; 0.50–0.87 and HR: 0.74; 0.57–0.95, respectively). At the end point of follow-up, the insulinogenic index (IGI), but not the insulin sensitivity index (ISI), differed among the groups. Alcohol consumption pattern had a J-shaped association with the incident type 2 diabetes in Korean men. The IGI showed an inverted J-shaped association according to alcohol drinking pattern, but the ISI was not a J-shape.

## Introduction

The prevalence of type 2 diabetes (T2D) in Korea has increased as rapidly as in Western countries for about two decades. To date, several factors (including obesity) have been reported to affect disease onset. One such factor is alcohol consumption, which is harmful to public health in Korea. More than 70% of Korean men are current drinkers and tend to undertake drinking binges; >50% of drinkers drink more than 60 g at a single sitting^1^. Therefore, alcohol consumption is an important risk factor for non-communicable diseases, including T2D, in Korea.

Moderate consumption of alcohol might have a beneficial effect on glucose metabolism and T2D prevention^2–6^. The relationship between alcohol consumption and T2D incidence is U- or J-shaped in both sexes. Jee *et al*.^7^ suggested that 1–24 g/day alcohol intake reduced the diabetes incidence risk compared with non-drinkers in Korean men and women. However, the beneficial effect of alcohol on diabetes remains controversial because several other studies have shown that moderate alcohol consumption is not associated with a reduction in the risk of T2D onset^8–11^.

This discrepancy might be due to the heterogeneity of study participants. Many studies have yielded different results depending on sex and race, likely due to the effects of different confounders and genetic mechanisms regulating alcohol intake and metabolism. In a recent meta-analysis, Craig *et al*.^12^ showed that the reduction in risk among moderate alcohol drinkers may be confined to women and may have been overestimated. In addition, they suggested that moderate drinking in Asian populations has no protective effect on T2D incidence. Another cause of the discrepancy might be a bias in the method of alcohol intake measurement. In most studies, average alcohol intake has been measured at one timepoint, and intake has been assumed to be stable over time. However, alcohol consumption is dynamic, especially over longer periods; therefore, measurement at a single timepoint could confound the results.

In the present study, we investigated the association of alcohol intake with T2D incidence in men using 12-year follow up data from a Korean adult cohort from the Ansan-Ansung study. To assess the alcohol consumption status of participants, we categorized them into four groups according to alcohol consumption pattern during the follow-up period and analyzed hazard ratios (HRs) for incident T2D. We also investigated the involvement of β-cell function and insulin sensitivity in the development of T2D.

## Results

The baseline characteristics of study participants by the over 10-years alcohol consumption pattern and baseline daily alcohol consumption presented in Table 1 and Supplementary Table S1, respectively. Diastolic blood pressure, triglycerides, HDL cholesterol, AST, and gamma-glutamyl transpeptidase (γ-GTP) levels differed among groups according to both categorizations (*p* < 0.05), but systolic blood pressure and ALT level differed significantly only among groups classified by the 10-years consumption pattern (*p* < 0.05).

**Table 1:** Baseline characteristics of participants (alcohol consumption pattern categorization).

	Alcohol consumption pattern				P-value^3^
	Never-drinking	Light	moderate	Heavy	
Number of subject(%)	338(19.4)	462(26.1)	666(37.6)	306(17.2)	
Age	52.8 ± 8.4^a^	51.2 ± 8.2^b^	49.3 ± 7.7^c^	49.0 ± 7.9^c^	<0.001
Body mass index	24.1 ± 3.0^a^	24.3 ± 2.8^a^	24.3 ± 2.8^a^	24.5 ± 3.0^a^	0.500
Systolic blood pressure(mmHg)	119.6 ± 16.7^ab^	118.0 ± 16.4^b^	120.0 ± 17.2^ab^	121.8 ± 16.9^a^	0.024
Diastolic blood pressure(mmHg)	79.9 ± 11.4^b^	79.9 ± 10.9^b^	81.7 ± 12.0^a^	82.5 ± 11.4^a^	0.002
Triglycerides(mg/dL)	153.4 ± 98.4^b^	153.1 ± 103.2^b^	169.4 ± 128.3^b^	186.5 ± 140.1^a^	<0.001
HDL-cholesterol(mg/dL)	44.2 ± 9.6^c^	45.1 ± 9.8^c^	49.1 ± 11.4^b^	50.7 ± 11.7^a^	<0.001
AST(IU/L)	26.8 ± 15.5^bc^	25.0 ± 8.9^c^	28.3 ± 15.3^b^	31.9 ± 15.6^a^	<0.001
ALT(IU/L)	29.9 ± 31.9^ab^	26.1 ± 15.5^b^	29.9 ± 31.6^ab^	32.1 ± 21.8^a^	0.018
Total cholesterol(mg/dL)	197.3 ± 33.8^a^	196.4 ± 33.4^a^	201.4 ± 33.5^a^	198.5 ± 39.0^a^	0.085
Γ-GTP	31.1 ± 25.2^c^	31.2 ± 26.6^c^	53.9 ± 85.1^b^	78.4 ± 82.1^a^	<0.001
IGI_60_^1^	6.2(5.3–7.2)^ab^	7.2(6.2–8.2)^a^	6.3(5.6–7.0)^ab^	5.3(4.4–6.3)^b^	0.067
ISI^2^	9.7(9.0–10.4)^a^	9.5(8.9–10.0)^a^	9.2(8.8–9.6)^a^	10.0(9.3–10.7)^a^	0.240

All data except β-cell function and insulin sensitivity are represented as mean ± standard deviation (SD). ^1^Insulin secretion refers to the insulinogenic index (IGI_60_) and is shown as the geometric mean (95% confidence interval, CI). ^2^Insulin sensitivity refers to the Matsuda index (ISI) and is shown as the geometric mean (95% confidence interval, CI). The participants were categorized into four groups based on follow-up measurement (consumption pattern). ^3^p-values were determined using one-way anova and post-hoc (Duncan) for continuous variables in according to alcohol consumption pattern at baseline and over 10-years. ^a,b,c,d^Different letters indicate significant difference of means among four groups by Duncan test.

We first investigated the relationship between baseline drinking status (Table 2) and incident diabetes. No reduction in risk was observed in the <5 g/day and ≥5, <30 g/day alcohol intake groups compared with the non-drinking group. The adjusted HR (95% CI) for those drinking <5 g/day was 0.96 (0.71–1.30), and that for those drinking ≥5, <30 g/day was 1.02 (0.81–1.30). The ≥30 g/day consumption group had an increased risk of diabetes [HR 1.42 (1.10–1.85)].

**Table 2:** Association between alcohol consumption at baseline and 12-year incidence of type 2 diabetes.

	Hazard Ratio(95% CI)			
	Non-drinking	<5 g/day	≥5, <30 g/day	≥30 g/day
	139(28.0%)	67(23.8%)	170(26.4%)	110(34.5%)
Model 1	Ref	0.93(0.70–1.24)	1.02(0.82–1.27)	1.42(1.12–1.81)
Model 2	Ref	1.03(0.77–1.38)	1.03(0.82–1.29)	1.42(1.12–1.83)
Model 3	Ref	0.96(0.71–1.30)	1.02(0.81–1.30)	1.42(1.10–1.85)

The participants were categorized into four groups based on the baseline measurement (one-point measure). Model 1: adjusted for age and Body mass index. Model 2: adjusted for age, Body mass index, family history of diabetes, and smoking. Model 3: adjusted for age, Body mass index, family history of diabetes, smoking, physical activity, total energy intake and IGI_60_.

**Figure 1:**
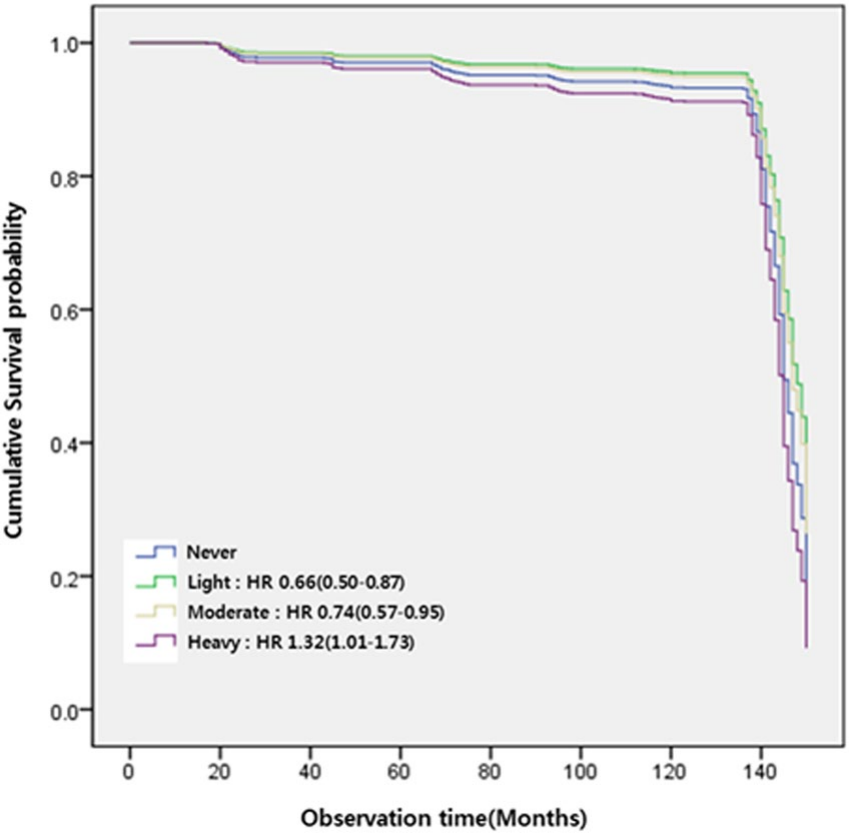
Kaplan-Meier curve of the incidence of type 2 diabetes by alcohol consumption pattern.

Drinking status during the follow-up period was categorized by drinking pattern. Kaplan-Meier curves and adjusted HR were presented in Fig. 1 and Table 3. Alcohol had a beneficial effect on incident diabetes in participants who maintained light and moderate alcohol consumption patterns during follow-up (p < 0.001). The adjusted HR (95% CI) for the light drinking group was 0.66 (0.50–0.87) and that for the moderate drinking group was 0.74 (0.57–0.95). The heavy consumption group also had an increased risk of incident diabetes; the adjusted HR for this group was 1.32 (1.01–1.73).

**Table 3:** Association between alcohol consumption pattern during follow-up period and 12-year incidence of type 2 diabetes.

	Hazard Ratio(95% CI)			
	Never-drinking	Light	Moderate	Heavy
	119(35.2%)	95(20.6%)	155(23.3%)	117(38.2%)
Model 1	Ref	0.64(0.49–0.84)	0.749(0.59–0.96)	1.40(1.08–1.82)
Model 2	Ref	0.68(0.52–0.89)	0.78(0.61–1.01)	1.38(1.05–1.80)
Model 3	ref	0.66(0.50–0.87)	0.74(0.57–0.95)	1.32(1.01–1.73)

The participants were categorized into four groups based on follow-up measurement (consumption pattern). Model 1: adjusted for age and Body mass index. Model 2: adjusted for age, Body mass index, family history of diabetes, and smoking. Model 3: adjusted for age, Body mass index, family history of diabetes, smoking, physical activity, total energy intake and IGI_60_.

**Figure 2:**
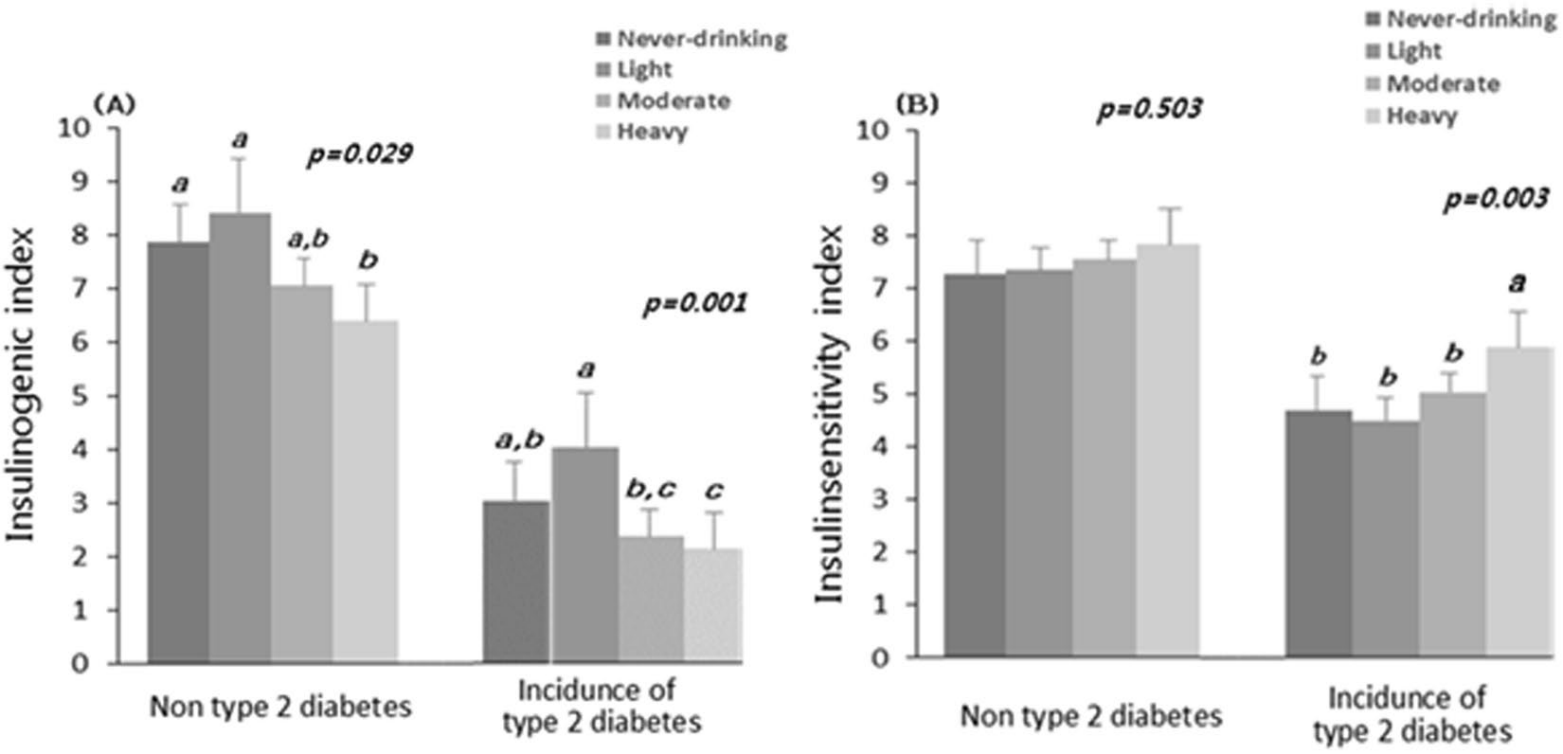
The associations between alcohol consumption pattern and (**A**) insulin secretion capacity (IGI_60)_ and (**B**) insulin sensitivity (ISI) at the end point of follow-up. **A**, and **B** is shown as the geometric mean and Error bars represent 95% CIs. *P*-values were determined using one-way anova and post-hoc (Duncan) for continuous variables according to alcohol consumption pattern at baseline and over 10-years. Different letters, a,b,c,d, indicate significant difference of means among four groups by Duncan test.

To determine the factor responsible for this effect, β-cell function and insulin sensitivity were analyzed. At baseline, the ISI did not differ among the groups, but the IGI_60_ differed among the incidence of type 2 diabetes (Supplementary Figure S1). However, at the end point of follow-up, the IGI_60_, but not the ISI, showed an inverted J-shaped association (Fig. 2). Importantly in the heavy drinking group, the IGI_60_ was significantly lower than those of other groups but not the ISI (Fig. 2A). Only in the heavy drinkers who had been diagnosed with type 2 diabetes, there was an increase of insulin sensitivity (Fig. 2B), which means that long-term heavy drinking might be able to slightly improve insulin sensitivity but reduction of insulin secretion in this group could more impact on the incidence of type 2 diabetes. Therefore these results suggests that insulin secretion capability, not insulin sensitivity, is a major factor responsible for alcohol-related diabetes onset.

## Discussion

In the present study, we found a J-shaped association between the level of regular alcohol consumption and incident diabetes in Korean men. To our knowledge, this study is the first to examine the relationship between alcohol consumption and diabetes in Korean men that involved long-term follow-up of alcohol intake.

The association of alcohol consumption with T2D has been examined previously in multiple studies. A 2016 meta-analysis by Li *et al*.^4^ included 26 cohort studies with 31,621 T2D cases. The authors showed that moderate alcohol consumption reduced the risk ratio to 0.8 (0.72, 0.89) compared with low alcohol consumption in men. However, this beneficial effect of alcohol on incident diabetes was not detected in four studies of Asian populations (in Korea and Japan)^13–16^. In this study, we also found no beneficial effect of moderate alcohol consumption on diabetes development when the alcohol consumption measured at baseline (one-point measure).

In most prospective studies, alcohol consumption has been measured at only one timepoint, which entails the assumption that intake is stable over time. However, alcohol consumption is dynamic and is influenced by health and socioeconomic status, which are important confounders. Therefore, we included only individuals with alcohol consumption data for the entire follow-up period and used the alcohol consumption pattern during the follow-up period to reduce this bias. As a result, a beneficial effect of alcohol was detected in the light and moderate drinking groups. Recently, Joosten *et al*.^17^ demonstrated that changes in alcohol consumption were associated with the risk of T2D. This finding suggests that a point measure of alcohol consumption is inappropriate for studies of the association between alcohol consumption and incident diabetes. In the present study, we also demonstrated that point and follow-up measurements of alcohol intake yielded different results regarding the association of alcohol consumption with incident diabetes. Therefore, the alcohol measurement method and categorization of participants according to alcohol consumption level are critical.

The association of alcohol consumption with a reduced risk of T2D has been explained in part by increased insulin sensitivity. However, the effect of moderate alcohol consumption on insulin sensitivity remains controversial. A recent meta-analysis showed that alcohol consumption did not influence insulin sensitivity.^3^ Moreover, alcohol intake tended to improve insulin sensitivity in women, but not in men. Our results showed that alcohol intake level does not affect insulin sensitivity, with the exception of the heavy alcohol consumption group, in Korean middle-aged men. However, pancreatic β-cell function, estimated by the IGI_60_, had an inverted J-shaped trend. Several recent studies have suggested that impaired β-cell function has a greater effect on the development of diabetes than does insulin resistance in Asian people^18–21^. We previously showed that heavy alcohol consumption was associated significantly with reduced insulin secretion, but not insulin sensitivity, in Koreans^22^. The mechanism of the effect of alcohol on insulin secretion is unclear, but Kim *et al*.^23^ found that chronic alcohol consumption potentiates the development of diabetes through pancreatic β-cell dysfunction. Therefore, the effect of drinking on incident diabetes in Korean men may be explained by its impact on β-cell function, rather than insulin sensitivity.

### Study Strengths and Weaknesses

The strengths of our study include its population-based design, prospective follow-up of alcohol consumption and incident diabetes, and inclusion of a broad range of confounding factors. Moreover, we compared the effect of alcohol on incident diabetes using two alcohol measurement methods. We also analyzed participants’ β-cell function and insulin sensitivity to investigate the mechanism underlying the effect of alcohol on incident diabetes. However, the limitations of our study include possible bias from self-reported drinking status. Self-reported alcohol consumption is generally considered to have acceptable reliability and validity for most research purposes.

Another limitation of this study is that it analyzed only male in the cohort population. We excluded women’s data from analysis because the majority of women in the cohort was never drinker or non-drinker (>80%) and there were insufficient numbers of drinking women to be analyzed statically. Therefore, it need to study the association of alcohol consumption and incidence of diabetes from other large population cohort.

Finally, alcohol has been known for a major risk factor of pancreatic diseases including chronic pancreatitis. It is the causative agent in nearly 50% of cases of chronic pancreatitis^24^ and increases the risk of this disease in a dose-dependent manner^25^. In addition, this disease can be a cause of diabetes which is classified as pancreatogenic diabetes or Type 3c diabetes^26, 27^. Therefore, we cannot rule out the possibility that alcohol-related pancreatitis may increase diabetes incidence in the heavy drinkers. This is an important issue for future research. However, the clinical data we used for our study did not include any information of pancreatic disease or type 3c diabetes. Further research should be undertaken to investigate the prevalence of type 3c diabetes in the heavy drinking population in Korean.

## Conclusions

Alcohol consumption pattern had a J-shaped association with the risk of T2D in Korean men. The IGI exhibited an inverted J-shaped association according to alcohol drinking pattern, but the ISI was not a J-shape. These findings suggest that the effects of alcohol on incident diabetes are associated with β-cell function, rather than insulin sensitivity. Importantly, the IGI of the heavy alcohol consumption group was significantly lower than that of the other groups, despite their normal glucose concentrations, suggesting that heavy drinking is an important risk factor in Korean men.

## Materials and Methods

### Study Population

The prospective, community-based Asan-Ansung Cohort Study has been described in detail previously^28^. The study is part of the Korean Genome and Epidemiology Study, a Korean government–funded epidemiological survey of trends in chronic diseases. The baseline survey was undertaken in 2001–2002 and follow-up examinations have been performed every 2 years. Data from 2001–2012 were included in this study. We used data from men aged 40–69 years who lived in urban Ansan or rural Ansung. Individuals with missing data regarding the incidence of T2D (*n* = 1507), and those with baseline T2D (*n* = 752) were excluded. About alcohol consumption, we excluded individuals who met at least one of the following criteria (*n* = 754); i) those who responded to <80% of follow-up questionnaires about alcohol consumption, ii) those who showed drastic changes in their alcohol consumption pattern during the follow-up (more than two level change in consecutive examines), iii) those who alcohol consumption information missing. A total of 1772 participants were included in this study. All participants provided written informed consent. Data were released from the National Biobank of Korea, the Centers for Disease Control and Prevention, Republic of Korea, and the study protocol was approved by the Korean National Institute of Health Institutional Review Board (2017-02-08-PE-A). All study protocols were carried out in accordance with approved guidelines.

### Alcohol Consumption Measurement

Information on alcohol consumption was collected at baseline and biennial follow-up examination by using an interview-based questionnaires. Participants were asked whether they had ever consumed at least 1 alcoholic drink every month, and if they had, they were asked whether they were former-drinkers or current-drinkers. In the case of current-drinkers, they were additionally asked to complete a questionnaire that inquired about the amount and frequency of alcohol consumed in the past 30 days. A total daily alcohol consumption was calculated using the average frequency, amount per occasion, and alcohol content of 1 standard drink. We classified the participants into four groups (non-drinking, <5 g/day, ≥5, <30 g/day, and ≥30 g/day) using baseline total daily alcohol consumption.

To better represent long-term alcohol consumption and to minimize the within-person variation, we created the pattern of alcohol consumption using a total daily alcohol consumption from baseline to before incidence of T2D. We classified the participants into four groups as follows: never-drinking, those who categorized ‘did not drink’ during the entire follow-up period; light, moderate, and heavy drinking, those who categorized ‘ <5 g/day’, ‘≥5, <30 g/day’, and ‘≥30 g/day’ in more than 60% of their follow-up period, respectively. In case the participants categorized the different consumption groups at same rate, we classified them into a less consumption level group, for example, if someone categorized ‘≥5, <30 g/day’ two times and ‘≥30 g/day’ two times, then was categorized as moderate drinking group.

### Incidence of Type 2 Diabetes

We performed the oral glucose tolerance test (OGTT, 75 g) in all participants. And T2D was estimated using the OGTT at each follow-up assessment. Incident diabetes was defined as fasting glucose level >126 mg/dL or 2-h post-OGTT glucose level >200 mg/dL. In addition, participants who reported current therapy with antidiabetic medication and insulin administration were considered to have T2D. The participants were followed until the development of T2D or the last examination.

### Potential Confounding Variables

Information on age, family history of diabetes, and smoking (pack-years) was obtained using questionnaires administered during interviews. Body mass index (BMI) was calculated as weight (in kilograms) divided by height (in meters) squared. Information on physical activity was obtained using a survey with an open-question about the hours spent in a typical day at following levels of intensity: low, medium, high. The amount of physical activity was classified as none or low, medium, and high intensity exercise. Each type of exercise was defined as ≥30 min/day. The fasting plasma concentrations of glucose, total cholesterol, triglycerides (TG), high-density lipoprotein (HDL) cholesterol, and alanine and aspartate aminotransferases (ALT and AST, respectively) were measured using a Hitachi 747 chemistry analyzer (Hitachi Ltd., Tokyo, Japan) following the manufacturer’s recommendations. Insulin was measured with an immunoradiometric assay kit (INS-IRMA Kit; Biosource, Nivelles, Belgium) using a gamma counter system (Packard Instrument Company, Meriden, CT, USA).

Pancreatic β-cell function and insulin sensitivity were estimated using 75-g OGTTs. Plasma was obtained at 0, 60, and 120 min after OGTT for measurement of plasma glucose and insulin concentrations. Plasma glucose concentrations were measured using the hexokinase method. Plasma insulin concentrations were measured as reported previously. The 60-min insulinogenic index (IGI_60_)^29^ was calculated using the following equation:

1 $$IG{I}_{60}=\frac{({{\rm{insulin}}}_{60}-{{\rm{insulin}}}_{0},\frac{{\rm{\mu }}{\rm{U}}}{{\rm{mL}}})}{({{\rm{glucose}}}_{60}-{{\rm{glucose}}}_{0},\frac{{\rm{mmol}}}{{\rm{L}}})}$$

insulin sensitivity was estimated using the OGTT (75 g) and Matsuda insulin sensitivity index (ISI) after 0, 60, and 120 min^30^. The ISI was calculated using the following equation:

2 $${\rm{ISI}}=\frac{10000}{\sqrt{({{\rm{glucose}}}_{0},\frac{{\rm{mg}}}{{\rm{dL}}})\times ({{\rm{insulin}}}_{0},\frac{{\rm{\mu }}{\rm{U}}}{{\rm{mL}}})\times (\mathrm{mean}\,\mathrm{glucose},\frac{{\rm{mg}}}{{\rm{dL}}})\times (\mathrm{mean}\,\mathrm{insulin},\frac{{\rm{\mu }}{\rm{U}}}{{\rm{mL}}})}}$$

### Statistical Analysis

Descriptive statistics [means ± standard deviations and geometric means with 95% confidence intervals (CIs)] were calculated to present the characteristics of the study population according to alcohol consumption at baseline and over 10 years. Analysis of variance (ANOVA) was used for comparison of continuous variables. ANOVA (with Duncan’s post hoc test) was conducted to compare clinical characteristics according to alcohol consumption at baseline and over 10 years. Chi-square tests were used for categorical variables. The IGI_60_ and ISI trajectories in the T2D and non-T2D groups were examined according to alcohol consumption pattern over 10 years. Cox regression analyses were performed to examine the associations of incident T2D risk with alcohol consumption at baseline and over 10 years. The association of alcohol consumption pattern with incident T2D risk was estimated after adjusting for age, BMI, family history of diabetes, smoking, physical activity, total energy intake and IGI_60_. Normal variables with non-Gaussian distributions were subjected to logarithmic transformation. All analyses were performed using SAS 9.4 (SAS Institute, Cary, NC, USA). Statistical tests were two sided, and *p* values <0.05 were considered to indicate statistical significance.

## Electronic supplementary material

Supplementary Information
