# Supplementary material for: Association between alcohol consumption pattern and the incidence risk of type 2 diabetes in Korean men: A 12-years follow-up study
Source: Sci Rep. 2017 Aug 4;7:7322. doi: 10.1038/s41598-017-07549-2 (PMC5544746; doi:10.1038/s41598-017-07549-2)
Supplement: Supplementary file 1 — Supplementary Information [file 41598_2017_7549_MOESM1_ESM.doc]

Association between alcohol consumption pattern and the incidence risk of type 2 diabetes in Korean men: A 12-years follow-up study

Dae-Yeon Lee^1,2,†^, Min-Gyu Yoo^1,†^, Hyo-Jin Kim^1^, Han Byul Jang^1^, Jae-Hong Kim^2^, Hye-Ja Lee^1,*^, Sang Ick Park^1,*^

|  | Alcohol consumption at baseline^3^ | | | | P-value^4^ |
| --- | --- | --- | --- | --- | --- |
|  | Non-drinker | >5 g/day | ≥5, < 30 g/day | ≥30 g/day |  |
| Number of subject(%) | 496(28.5) | 281(16.2) | 643(37.0) | 319(18.3) |  |
| Age | 52.0±8.3^a^ | 51.5±7.9^a^ | 49.4±7.9^b^ | 48.9±7.8^b^ | <0.001 |
| Body mass index | 24.2±2.9^a^ | 24.2±2.8^a^ | 24.4±2.8^a^ | 24.4±2.9^a^ | 0.500 |
| Systolic blood pressure(mmHg) | 119.0±16.1^a^ | 118.1±17.3^a^ | 120.5±17.5^a^ | 120.6±16.2^a^ | 0.129 |
| Diastolic blood pressure(mmHg) | 79.9±11.1^b^ | 79.8±11.3^b^ | 81.8±11.8^a^ | 82.3±11.7^a^ | 0.003 |
| Triglycerides(mg/dL) | 148.5±91.5^b^ | 156.1±105.6^b^ | 174.4±133.0^a^ | 179.4±137.0^a^ | 0.002 |
| HDL-cholesterol(mg/dL) | 44.0±9.3^d^ | 46.0±10.3^c^ | 48.8±11.2^b^ | 50.9±12.0^a^ | <0.001 |
| AST(IU/L) | 26.1±13.8^c^ | 25.3±8.6^c^ | 28.7±15.7^b^ | 30.7±14.9^a^ | <0.001 |
| ALT(IU/L) | 28.5±27.7^a^ | 27.3±16.8^a^ | 30.2±32.0^a^ | 30.5±20.3^a^ | 0.328 |
| Total cholesterol(mg/dL) | 195.9±33.7^a^ | 197.9±33.2^a^ | 201.0±34.4^a^ | 199.7±37.0^a^ | 0.084 |
| Γ-GTP | 30.1±25.7^c^ | 33.6±26.9^c^ | 55.3±88.4^b^ | 72.5±74.9^a^ | <0.001 |
| IGI_60_^1^ | 6.7(5.9-7.6)^a^ | 6.5(5.5-7.7)^ab^ | 6.5(5.8-7.3)^ab^ | 5.2(4.4-6.2)^a^ | 0.109 |
| ISI^2^ | 9.7(9.2-10.3)^ab^ | 9.3(8.7-9.9)^a^ | 9.2(8.8-9.6)^ab^ | 10.0(9.4-10.7)^b^ | 0.119 |
| Pre-diabetes | 140(28.2%) | 87(31.0%) | 191(29.7%) | 119(37.3%) | 0.042^5^ |

**Supplementary Table S1. General characteristics of Korean men at baseline. All data except β-cell function and insulin sensitivity are represented as mean ± standard deviation (SD).** ^1^ Insulin secretion refers to the insulinogenic index (IGI_60_) and is shown as the geometric mean (95% confidence interval, CI). ^2^ Insulin sensitivity refers to the Matsuda index (ISI) and is shown as the geometric mean (95% confidence interval, CI). ^3^ alcohol consumption was categorized as follows: Non-drinker were individuals who did not consume alcohol; <5g/day; ≥5, < 30g/day; and ≥30g/day. ^4^ p-values were determined using one-way anova and post-hoc (Duncan) for continuous variables in according to alcohol consumption at baseline. ^a,b,c,d^ Different letters indicate significant difference of means among four groups by Duncan test. ^5^ Chi-square tests were used for categorical variables by alcohol consumption pattern.


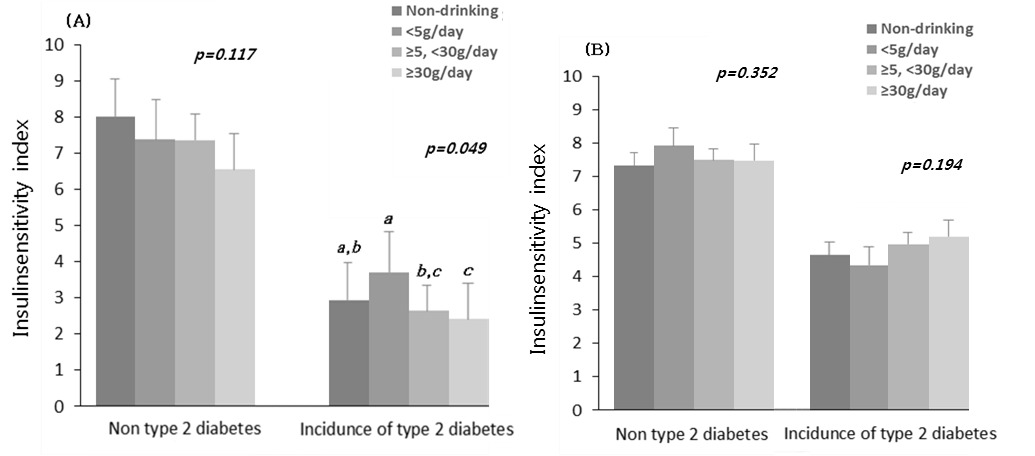


**Supplementary Figure S1. The associations between alcohol consumption pattern at baseline and (A) insulin secretion capacity (IGI_60_) and (B) insulin sensitivity (ISI) at the end point of follow-up.** A and B is shown as the geometric mean and Error bars represent 95% CIs. P-values were determined using one-way anova and post-hoc (Duncan) for continuous variables according to alcohol consumption pattern at baseline. a,b,c,d Different letters indicate significant difference of means among four groups by Duncan test.
